# Effect of focused power ultrasound-mediated perirenal fat modification on primary hypertension: protocol of a multicenter, randomized, double-blinded, sham-controlled study

**DOI:** 10.1186/s13063-023-07249-5

**Published:** 2023-03-23

**Authors:** Menghuan Li, Jing Shi, Yanhui Sheng, Yuqing Zhang, Tingting Wu, Jiaming Yang, Kerui Zhang, Wei Sun, Xiangqing Kong

**Affiliations:** 1 Department of Cardiology, the First Affiliated Hospital of Nanjing Medical University, No.300 Guangzhou Road, Nanjing, 210000 China; 2 Gusu School, Nanjing Medical University, Suzhou, 215100 China; 3 Department of Cardiology, the Affiliated Jiangning Hospital of Nanjing Medical University, Nanjing, 210000 China

**Keywords:** Hypertension, Blood pressure, Perirenal fat, Focused power ultrasound, Therapy, Randomized controlled trial

## Abstract

**Background:**

Perirenal fat plays a key role in sustaining pathological high blood pressure. We aim to investigate the efficacy of intervention for perirenal fat mediated by focused power ultrasound (FPU) on primary hypertension.

**Methods:**

A multicenter, randomized, sham-controlled, double-blinded trial will be implemented in 200 participants with mild to moderate hypertension. All enrolled participants will be randomly allocated to perirenal fat modification (PFM) intervention using FPU or sham-procedure at a ratio of 1:1 and will be followed up at 24 h, 14 days, 30 days, and 90 days after the intervention. The primary endpoint is changes in office systolic blood pressure (SBP) at 30 days compared with baseline. The secondary endpoints include the changes in office SBP from baseline to 90 days, changes in 24-h mean SBP from baseline to 30 days and 90 days, and changes in heart rate from baseline to 30 days. Safety endpoint is defined as any severe adverse events related to the intervention.

**Discussion:**

The present study is the first to use noninvasive FPU to intervene in perirenal fat to achieve the goal of reducing blood pressure for patients with essential hypertension. Our study is expected to provide a new treatment strategy to control high blood pressure.

**Trial registration:**

ClinicalTrials.gov. NCT05049096. Registered on September 7, 2021.

Protocol version: Version 1.3.1, data 23 August 2021.

Sponsor: Prof. Xiangqing Kong is the principal investigator of this trial.

## Administrative information

**Table:** 

Title {1}	Effect of focused power ultrasound-mediated perirenal fat modification on primary hypertension: protocol of a multicenter, randomized, double-blinded, sham-controlled study
Trial registration {2a and 2b}.	ClinicalTrials.gov Identifier: NCT05049096. Registered on September 7, 2021.https://clinicaltrials.gov/ct2/show/NCT05049096?term=05049096&draw=2&rank=1
Protocol version {3}	Version 1.3.1, data 23 August 2021
Funding {4}	This work was funded by the National Natural Science Foundation of China (No.82150002, No.81627802) and the Science Foundation of Gusu School (No. GSKY20220102).
Author details {5a}	1. Menghuan Li, M.D., The First Affiliated Hospital of Nanjing Medical University, Nanjing, China. 2. Jing Shi, Ph.D., The First Affiliated Hospital of Nanjing Medical University, Nanjing, Jiangsu, China. 3. Yanhui Sheng, Ph.D., The First Affiliated Hospital of Nanjing Medical University, Nanjing, Jiangsu, China; Gusu School, Nanjing Medical University, Suzhou, Jiangsu, China. 4. Yuqing Zhang, M.D., The Affiliated Jiangning Hospital of Nanjing Medical University, Nanjing, Jiangsu, China. 5. Tinting Wu, M.D., The First Affiliated Hospital of Nanjing Medical University, Nanjing, Jiangsu, China. 6. Jiaming Yang, M.D., The First Affiliated Hospital of Nanjing Medical University, Nanjing, Jiangsu, China. 7. Kerui Zhang, M.D., Gusu School, Nanjing Medical University, Suzhou, Jiangsu, China. 8. Wei Sun, M.D., Ph.D., The First Affiliated Hospital of Nanjing Medical University, Nanjing, Jiangsu, China. 9. Xiangqing Kong, M.D., Ph.D., The First Affiliated Hospital of Nanjing Medical University, Nanjing, Jiangsu, China; Gusu School, Nanjing Medical University, Suzhou, Jiangsu, China.
Name and contact information for the trial sponsor {5b}	Prof. Xiangqing Kong (principal investigator), The First Affiliated Hospital of Nanjing Medical University, Nanjing, Jiangsu, China; Email: kongxq@njmu.edu.cn. Phone: 02568303130. Contact for public and scientific queries: Wei Sun, Email: shunwee@126.com.
Role of sponsor {5c}	Prof. Xiangqing Kong serves as the principal investigator of this trial and applied for scientific financial support. The funders played no role in the study design, implementation, data analysis, and results publication.

## Introduction

### Background and rationale

Hypertension is one of the most common cardiovascular diseases, whose prevalence exceeds 30% adult population worldwide, and has become the leading cause of global health issues and economic burden [1, 2]. Blood pressure (BP) is deemed to be related to cardiovascular risk. Effective BP management is critical to decreasing the incidence of stroke, heart attack, and heart failure [3–5]. However, the rate of awareness, treatment, and control of hypertension were low, especially in developing countries [5, 6]. The control rate of hypertension in China was even as low as 5.7%, raising great concerns about the health of Chinese individuals [6].

Although pharmacological antihypertensive therapies are the main measures to reduce BP, the percentage of hypertensive patients whose BP attain the target guideline recommendation is unacceptably poor. Exploration of other options to reduce BP is urgent. Visceral fat is closely related to the occurrence of hypertension. Notably, perirenal fat is a unique connected tissue that is well vascularized and innervated [7]. Cumulating evidence showed that perirenal adipose tissue (PRAT) was significantly associated with high BP [7–9] Recently, it was revealed that perirenal adipose afferent nerves played a key role in sustaining pathological high BP in rats [10]. Long-term reduction of BP was observed in spontaneously hypertensive rats by bilateral PRAT ablation [10]. Thus, we hypothesize that the PRAT can be a potential target for lowing BP in clinical practice.

Focused ultrasound is increasingly used in the broad area of medicine, especially in the ablation of solid tissue, such as tumors [11]. This promising treatment could effectively ablate the target area in a non-invasive way with superb precision of energy delivery [12, 13].

Herein, we conducted a multicenter, randomized, sham-controlled, double-blinded study using focused ultrasound to modify the PRAT to explore the efficacy of this kind of modification for the treatment of essential hypertension.

### Objectives

This study is mainly to assess the efficacy of perirenal fat modification (PFM) therapy with focused ultrasound for hypertensive patients with uncontrolled blood pressure. In addition, the occurrence of adverse events (e.g., renal injury, intestinal perforation) will be recorded to assess the safety outcomes.

### Trial design

This is an investigator-initiated, multicentered, randomized, sham-controlled, double-blinded, and exploratory clinical trial with a 3-month duration, which primarily aims to assess whether PFM therapy can reduce blood pressure in patients with grade one to two hypertension.

Eligible participants will be randomized at a ratio of 1:1 into an intervention group and a control group. The participants in the intervention group will receive PFM therapy with focused ultrasound, while a sham procedure will be performed for the participants assigned to the control group. All participants will be assessed for efficacy and safety at 24 h, 2 weeks, 1 month, and 3 months after PFM or sham procedure. The main indicators to evaluate efficacy are the changes in office BP and 24-h ambulatory BP. Adverse events will be recorded at each visit. Blood samples and urine samples will be collected, and imaging examinations will be performed at baseline, 24 h, 1 month, and 3 months. Participants are required to measure their office BP at a 2-week visit. Except for the operator who is responsible for focused ultrasound, all staff, including researchers, statisticians, and participants, are blinded to the groups of randomizations.

This protocol report follows the SPIRIT reporting guidelines [14].

## Methods: participants, intervention, and outcomes

### Study setting

This trial is initiated by the Cardiology Department, The First Affiliated Hospital of Nanjing Medical University. Eligible participants with uncontrolled BP will be recruited at three implementation sites, including the First Affiliated Hospital of Nanjing Medical University (Jiangsu Province), Nanjing Jiangning Hospital (Jiangsu Province), and Suzhou Municipal Hospital (Jiangsu Province).

### Eligibility criteria

The inclusive criteria are as follows:

• Individuals aged 18–65 years old
• Individuals have uncontrolled BP when receiving a medication regimen of one, two, or three antihypertensive medication classes for at least 4 weeks; and uncontrolled BP is defined as office systolic blood pressure (SBP) ≥ 140 mmHg and < 180 mmHg, and 24-h ambulatory BP monitoring (ABPM) average SBP ≥ 135 mmHg, regardless of diastolic BP
• Individuals have at least 20 mm in the anteroposterior, transverse, and axial diameters of inferior perirenal fat measured by ultrasound
• Individuals are willing to sign the informed consent of the study

The exclusive criteria are as follows:

• Individuals are diagnosed with secondary hypertension (e.g., renal parenchymal hypertension, renal artery stenosis, primary aldosteronism, pheochromocytoma, Cushing’s syndrome, aortic coarctation, severe obstructive sleep apnea-hypopnea syndrome)
• Individuals have a history of kidney and or kidney surrounding tissue surgery
• Individuals have impairment of liver or kidney function (ALT, AST, or creatinine is greater than 3 times the upper limit of normal reference)
• Individuals have a myocardial infarction, unstable angina pectoris, cerebrovascular accident, or transient ischemic attack within 6 months before enrollment
• Individuals have type 1 diabetes or poorly-controlled type 2 diabetes
• Individuals have uncontrolled thyroid dysfunction
• Individuals have urinary calculi and/or hematuria
• Individuals have atrial fibrillation
• Individuals have severe structural heart disease (e.g., heart valve disease, cardiomyopathy, congenital heart disease)
• Individuals have second-degree and above atrioventricular block
• Individuals have abnormal coagulation function
• Individuals have infected waist skin
• Individuals have a malignant tumor
• Individuals are pregnant, nursing, or planning to be pregnant
• Individuals are unwilling to sign informed consent
• Individuals fail to complete the screening period

### Intervention

Eligible patients will undergo either PFM with focused ultrasound or a sham procedure. It is expected that their prescribed antihypertensive medications should be unchanged in the 3 months follow-up period after the intervention.

### PFM procedure

Patients who are assigned to the PFM group will be placed in a lateral position on the treatment bed. Firstly, an ultrasonic probe is used to locate the rough position of inferior perirenal fat and the location needs to be marked on the waist skin. Secondly, a focused power ultrasonic probe is applied to determine the precise target area of perirenal fat. Sequentially, automatic physique measurement starts to calculate the optimal output power for individuals. Lastly, treatment initiates. Bilateral inferior perirenal fat shares the same procedure. The training sessions including recognition of inferior perirenal fat from ultrasonic images and operation of the equipment have been conducted at each study site by only one designated operator.

### Sham procedure

Patients who are assigned to the sham control group share the same procedure as PFM does except that the focused ultrasound will not transmit energy.

### Criteria for discontinuing

Participants may discontinue this trial for any of the following reasons:

• They may choose to withdraw for any reason
• They have severe side effects that require unmarking to get cured
• Their blood pressure is within “escape criteria,” defined as office SBP ≥ 180mmHg or < 90 mmHg
• They have a significant deviation from the study algorithm, e.g., changing the established antihypertensive drugs scheme
• Any other rational reasons to withdraw from the study

### Strategies to improve adherence

Before enrollment, we repeatedly confirm the participants’ willingness and compliance with the study’s requirements, especially drug numbers and doses, and being able to follow up within the next 3 months. We make efforts to monitor the adherence to drug use which is prescribed before allocation for at least 4 weeks. Blood and urine samples will be collected to measure the concentration of the antihypertensive drugs (diuretic, calcium channel blocker (CCB), angiotensin-converting enzyme inhibitors (ACEI)/angiotensin receptor blocker (ARB), beta-blocker, and alpha-blocker) at baseline, 1 month, and 3 months.

### Relevant concomitant care and interventions

Though lifestyle modification should be recommended for all hypertensive patients according to the hypertension guidelines, we asked recruited participants to maintain their current lifestyle, including physical activities, diet, and regular rest habits. In order to assess the changes in lifestyle, weight, waist circumference, or hip circumference will be recorded at each follow-up time.

### Outcomes

We speculate that PFM will have a significant blood pressure lowering effects compared with the sham-control group. The endpoints in the present study include primary endpoints, secondary endpoints, and safety endpoints.

Primary endpoint measure:

• Changes in office systolic blood pressure at 1-month compared with baseline

Secondary outcome measures:

• Changes in office blood pressure at 3 months compared with baseline
• Changes in mean systolic blood pressure measured by 24-h ambulatory blood pressure monitoring at 1 month compared with baseline
• Changes in mean systolic blood pressure measured by 24-h ambulatory blood pressure monitoring at 3 months compared with baseline
• Changes in the heart rate at 1 month compared with baseline
• Changes in the mean heart rate measured by 24-h ambulatory blood pressure monitoring at 1 month compared with baseline

Safety endpoint measures:

• Any severe adverse events (SAE) related to the intervention. The SAE was defined as acute renal failure, acute intestinal perforation, and thromboembolic events, etc.

### Participant timeline

The participant timeline is shown in Table 1. Briefly, all participants will undergo either PFM or sham-control therapy during 14 days screening period. They will be asked to follow up at 1 day, 14 days, 30 days, and 90 days after randomization.

**Table 1:** The schedule of enrolment, interventions, and assessments

	Study period					
	Enrolment	Allocation	Post-allocation			Close-out
Timepoint	*− 14 days*	Day 0	*Day 1*	*Day 14*	*Day 30*	*Day 90*
Enrolment:						
Eligibility screen	X					
Informed consent	X					
Interventions:						
* PFM*		X				
* Sham-control*		X				
Assessments:						
Office BP measurement	X		X	X	X	X
24-h ambulatory BP measurement	X		X		X	X
History of diseases	X					
Physical examinations	X				X	X
Drugs use record	X			X	X	X
Antihypertensive drugs concentration tests	X				X	X
Blood routine	X		X		X	X
Biochemical test	X		X		X	X
C-reactive protein	X		X			
Biomarkers of acute renal injury	X		X			
ECG	X		X		X	X
Renal and renal artery ultrasound	X		X		X	X
Carotid ultrasound	X		X		X	X
Cardiac ultrasound	X					
MR imaging of perirenal fat	X				X	X
Functional MR imaging of hypothalamus	X				X	X

*PFM*, perirenal fat modification. *BP*, blood pressure, *ECG*, electrocardiogram *MR*, magnetic resonance

### Sample size

The PASS software (version 2011) was used to calculate the sample size. This study is a parallel group design, whose primary endpoint is the difference in office BP at 1 month compared with baseline. Our previous pilot study was a single-arm design recruiting 15 participants with mild-moderate hypertension for PFM therapy with results unpublished. Based on the outcome of the pilot study, the mean change in office SBP from baseline to 1 month was expected to 10 mmHg with a deviation of 13 mmHg in the PFM group. Meanwhile, we assumed that a mean difference of office SBP was 5 mmHg [15, 16] with a deviation of 13 mmHg in the sham control group. The sample size was calculated as 100 in each group with a power of 80%, a two-sided significance level of 5%, and a drop-out rate of 15%.

### Recruitment

Potential eligible participants will be screened at the outpatient clinics of each study center. Advertisements of the trial introduction will be posted onsite at the hospital and on social media platforms.

### Assignment of interventions: allocation

#### Sequence generation

The randomized allocation sequence was generated at a 1:1 ratio in a completely random design among 200 participants by a statistician via the SAS 9.4 software (SAS Institute, Cary NC, USA) and integrated with a central computerized randomization system (Biomed Information Technology Co., Ltd. Beijing).

#### Concealment mechanism

The randomization codes were generated by a statistician and embedded in the computerized randomization system. The main investigators will not have permission to view the allocation except for the principal investigator of each study site and operators who are responsible for PFM operation. The principal investigators are only authorized for urgent unblinding when a serious complication related to the PFM procedure occurs.

#### Implementation

An independent randomization system account is created for investigators at each study site for the randomization implementation. Each investigator will log in to the system via a website to enter the randomization page, input the information of the participants, check the name and code of the study site, and confirm the randomization information. Then, the randomization code and allocated arm of the participant will be generated in the system background which is blinded for the investigators. Additionally, the operator at each study site will log in to the system using an independent account with the authority to view the allocated arms. At this time, the participants are successfully enrolled in this study.

#### Blinding

Study participants and all staff, including investigators, clinical care providers, statisticians, and personnel who recruit, follow-up with participants, and collect data are blinded to the randomizations. The operators will be unblinded to implement the PFM procedure and required to keep blinded to all other staff. After completing the procedure, we will ask about the participants’ feelings or any discomforts and if they can tell the real intervention and sham intervention. Once severe adverse events (SAE) that might be related to the PFM procedure occur, the principal investigators will be able to disclose the arm allocation. The disclosure reason, date, and location will be recorded in detail.

## Methods: data collection, management, and analysis

### Data collection

The outcomes will be assessed at baseline, 1 day, 14 days, 30 days, and 90 days after the intervention. All staff involved in the collection of data were trained by the standard operating procedure of the study. The data will be recorded utilizing electronic CRFs and EDC systems.

### Baseline data collection

The following measures will be completed at baseline.

• Written informed consent
• Clinical questionnaire
• Office BP and 24-h ambulatory BP
• Anthropometric information including height, weight, waist circumference, and hip circumference
• Electrocardiography and cardiac ultrasound
• Arteries stiffness assessments (PWV)
• Imaging examinations including ultrasonic examinations of renal and perirenal fat, carotid ultrasound, and magnetic resonance imaging for perirenal fat and hypothalamic function
• Blood tests including routine blood tests, blood biochemical tests, and C-reactive protein
• Urine tests including routine urine tests, urine biomarkers of acute renal injury (Ngal, TIMP2, IGFBP7)
• Hypertensive drug concentration detection (serum and urine)

### Data collection at 1 day after intervention

• Office BP and 24-h ambulatory BP
• Electrocardiography
• Arteries stiffness assessments (PWV)
• Imaging examinations including ultrasonic examinations of renal and perirenal fat and carotid ultrasound
• Blood tests including routine blood tests, blood biochemical tests, and C-reactive protein
• Urine tests including routine urine tests and urine biomarkers of acute renal injury (Ngal, TIMP2, IGFBP7)

### Data collection at 14 days after intervention

• Office BP

### Data collection at 30 days after intervention

• Office BP and 24-h ambulatory BP
• Anthropometric information including height, weight, waist circumference, and hip circumference
• Electrocardiography
• Arteries stiffness assessments (PWV)
• Imaging examinations including ultrasonic examinations of renal and perirenal fat, carotid ultrasound, and magnetic resonance imaging for perirenal fat and hypothalamic function
• Blood tests including routine blood tests, and biochemical blood tests
• Urine tests including routine urine tests
• Hypertensive drug concentration detection (serum and urine)

### Data collection at the final visit

• Office BP and 24-hour ambulatory BP
• Anthropometric information including height, weight, waist circumference, and hip circumference
• Electrocardiography
• Arteries stiffness assessments (PWV)
• Imaging examinations including ultrasonic examinations of renal and perirenal fat, carotid ultrasound, and magnetic resonance imaging for perirenal fat and hypothalamic function
• Blood tests including routine blood tests, and biochemical blood tests
• Urine tests including routine urine tests
• Hypertensive drug concentration detection (serum and urine)

All the eligible participants will have comprehensive health examinations and receive PFM therapy or sham-procedure for free. They will have close relationships with physicians who can provide professional consultations and medication guidance for their health problems. Traffic and accommodation subsidies will provide for all participants to promote participant retention and complete follow-up. If participants discontinue follow-up, the reasons will be recorded in detail, and their clinical treatment and other rights shall not be affected.

### Data management

Study data will be recorded via the Electronic Data Capture (EDC) system (Nanjing Yike Valtai Information Technology Co., Ltd). Well-trained practitioners will log into the website and input data. The EDC system has the function of automatically checking data format, rules, and range so as to ensure the accuracy of data. Moreover, manual reconfirmation by a third staff is also required. Any change in data recording is permitted and tracked with reasons and dates in the EDC system. Additionally, data quality will be under the supervision of a third party. All documents and data will be stored at the institutional office of each study site.

### Statistical method

For the primary endpoint, a mixed-effect model for repeated measures (MMRM) adjusted with baseline office SBP will be used to compare the group difference of the office SBP changes from baseline to 1 month. For the secondary endpoint, we will also use the MMRM method adjusted with mean SBP or heart rate to assess the changes in the mean SBP or heart rate from baseline to each visit point. And LS-means method adjusted with baseline SBP or heart rate will be used to estimate the changes in SBP or heart rate and 95% confidence intervals. Imputation for missing values of the main indicator will be conducted using the Markov chain Monte Carlo (MCMC) model which will be performed as the result of the sensitive analysis. We predefine an adverse event of laboratory index that is significantly abnormal at follow-up compared with baseline. Detailed presentation of adverse events will be described and explore the relationship with PFM products. The efficacy analyses will be carried out based on the full analysis set and the pre-protocol set. All baseline characteristics analysis will be conducted on the basis of the full analysis set. The safety evaluation will be analyzed from the safety analysis set. A two-sided *P* < 0.05 will indicate significance in all statistical analyses used by the SAS software V.9.4 (SAS Institute, Cary, NC).

An interim analysis is planned when 50 subjects (100 subjects in total) are enrolled in each group and completed 1 month follow-up, which will be conducted by an independent data monitoring committee (IDMC). The purposes of the interim analysis are as follows: (1) to evaluate the safety (a necessary disruption of the study is considered when there are serious or expected adverse events) and (2) to assess the efficacy. This trial will be expected to terminate in advance if the hypothesis test *P* value is less than the boundary value of the interim analyzed *α* for the primary endpoint. And if the *P* value is greater than interim analyzed *α* with statistical power less than 60%, re-estimating the sample size is needed to make the final statistical power greater than 80%. In accordance with O’Brien-Fleming method, the interim analyzed *α* = 0.005, and final *α* = 0.048.

### Methods: monitoring

#### Data monitoring

In order to monitor the trial process and participants’ safety, a trial steering committee (TSC) was established to make sure the trial run well. The members of this committee are experts from different professional fields, including hypertension management, cardiovascular disease, biostatistics and other fields. All personnel from the committee are absolutely independent from and have no competing interests with the funders and sponsor. We invited the staff from the Clinical Trial Institution of Jiangsu Province Hospital, and School of Public Health, Nanjing Medical University to regularly monitor the data and report to the committee. The TSC will meet onsite at each study site every three months to track the trial process and monitor the participant’s safety.

#### Harms and auditing

In this study, potential risks of PFM therapy are the thermal damage of local skin, subcutaneous tissue, and organs adjacent to perirenal fat. Any discomforts or abnormal indicators changes through energy emission path including skin, muscles, renal area, and intestine will be identified as adverse events (AEs) and will be recorded in eCRF and EDC system with details of onset time, severity, and duration. The severity of AEs and their correlations with PFM therapy will be carefully evaluated in accordance with the Common Terminology Criteria for Adverse Events [17]. Severe adverse events (SAEs) included deaths, life-threatening disease or injury, permanent damage to body structure or function, and diseases requiring hospitalization treatment that are determined by the investigator according to the principles of GCP. Investigators are responsible for dealing with the SAEs to protect the participants as much as possible and reporting the SAEs to the local ethics committee within 24 h. AEs will be reported within 3 days after each visit. All adverse events will be revealed in the trial publication. And the trial will be audited onsite periodically.

#### Ethics and dissemination

This study protocol was comprehensively considered and will not be amended easily without written permission from the Ethics Committee of the responsible unit. The revised protocol will be inspected by the local ethical boards again. The informed consent approved by local ethical boards will be presented to the potential participants and their authorized surrogates. Investigators is responsible for introducing the study design, procedure, potential harms, and benefits, and answering the participant’s questions. The participant can only be enrolled after signing their name on the page of the informed consent form. Additional sample collection is also noted on the pages of informed consent. For each visit, 5 ml blood samples and 2 ml urine samples will be collected and stored. To protect participants’ confidentiality, private information, including names, addresses, contact, and identified numbers will be kept within each investigation center. Access by any third party will be prohibited. All data used for analysis and report shall not have personal identifiers except for unique screening codes or randomization codes. Once the AEs occur, medical care will be provided for participants promptly. In addition, appropriate assistance and management will be presented according to the relevant laws and regulations.

The study results will be published in peer-review journals, presented at scientific conferences, and shared with all participants. Access to all deidentified data will be permitted only for certificated researchers with written approval from the responsible investigators. Any data required to support the protocol can be supplied on reasonable request.

## Discussion

Epidemiological studies have demonstrated that perirenal fat is closely associated with elevated blood pressure [8, 9]. It is reported that accumulation of fat in the renal sinus may promote hypertension due to compression of the lymphatic and venous vessels by perirenal fat, leading to activation of the renin-angiotensin-aldosterone system [18]. However, observational studies failed to clarify the pathological mechanism of hypertension caused by perirenal fat. Recently, researchers found that bilateral perirenal fat ablation leads to a long-term reduction of high blood pressure in spontaneously hypertensive rats and has no influence on normal blood pressure in control rats; they further indicate that perirenal afferent nerves serve as a pathological node of hypertension that sustains elevated blood pressure via suppressing CGRP, thereby being a potential therapeutic target to tackle primary hypertension [10]. And the perirenal fat is so distinguished that no other fat pad such as inguinal, pararenal, or epididymal adipose tissue is associated with the maintenance of high blood pressure in their research. Moreover, perirenal fat, especially the fat pad in the lower pole of the kidney, is a relatively independent solid tissue, which is different from other visceral fat, making it a potential target for clinical intervention to lower blood pressure.

Long-term use of medical treatment is the essential method for hypertensive patients, which is bound to have increased risks of adverse events. Especially for those who are resistant to antihypertensive drugs, traditional medical therapy is of little help. Therefore, novel therapeutic methods are urgent for lowing blood pressure, notably the instrument treatment for hypertension. Renal denervation (RDN) is an intervention that uses an ablation catheter to destruct sympathetic nerves of renal arteries, which might be a promising way to reduce blood pressure for individuals with resistant hypertension. Several randomized clinical trials with unblinded designs showed remarkable effects of RDN on lowering blood pressure and few complications [19, 20]. However, further RCT with a sham-control design presented a similar blood pressure reduction between the RDN group and the sham control group, indicating the placebo effects of RDN [21]. Even though a series of RCTs confirmed the effectiveness of RDN on elevated blood pressure after optimizing the criteria of the enrollment and working mode of the ablation catheter [22, 23], physicians are still concerned about the safety of RDN since it is an invasive procedure. Hence, a noninvasive way is needed to reduce blood pressure. Perirenal fat has become an ideal target for noninvasive intervention using focused power ultrasound. We previously conducted a small sample size study revealing that PFM therapy was a safe and effective way to lower blood pressure for individuals with essential hypertension [24].

Traditional focused power ultrasound has limited use on adipose tissue in the abdominal cavity. In order to equip a focused power ultrasound dedicated to the intervention of perirenal fat, we designed and developed a novel machine that can be effectively and safely used for the modification of perirenal fat. This novel FPU machine has the strength of temperature monitoring of the target area and personalized output power according to the fat characteristics of the subjects to ensure that the target perirenal fat can be effectively and safely modified.

To our knowledge, the present study is the first to use noninvasive FPU to intervene in perirenal fat to achieve the goal of reducing blood pressure for patients with essential hypertension. Our study is expected to provide a new treatment strategy to control high blood pressure. At the initial stage, we are exploring the efficacy of this new strategy for hypertensive patients on medication with mild to moderate elevated blood pressure. In the future, we will expand the population for adaption of this new therapy, such as new onset hypertensive patients, hypertensive patients without taking medications, and even refractory hypertensive patients.

## Trial status

This paper is in accordance with the protocol (version 1.3.1, data 23 August 2021). The study recruitment began on November 1, 2021, with 74 participants recruited by February 8, 2022, and is estimated to complete the study on September 30, 2023.

## Data Availability

The investigators will have access to the final trial data and material. Any other access to data without written approval from the responsible investigator is prohibited.
