# Correction: Effect of focused power ultrasound-mediated perirenal fat modification on primary hypertension: protocol of a multicenter, randomized, double-blinded, sham-controlled study

**DOI:** 10.1186/s13063-023-07343-8

**Published:** 2023-05-05

**Authors:** Menghuan Li, Jing Shi, Yanhui Sheng, Yuqing Zhang, Tingting Wu, Jiaming Yang, Kerui Zhang, Wei Sun, Xiangqing Kong

**Affiliations:** 1 Department of Cardiology, the First Afliated Hospital of Nanjing Medical University, No.300 Guangzhou Road, Nanjing, 210000 China; 2 Gusu School, Nanjing Medical University, Suzhou, 215100 China; 3 Department of Cardiology, the Afliated Jiangning Hospital of Nanjing Medical University, Nanjing, 210000 China

**Correction: BMC Trials 24, 221 (2023)**

**https://doi.org/10.1186/s13063-023-07249-5**

Following publication of the original article [1], we have been informed of a sample size calculation error. The deviation of 13 mmHg in the sample size section should be corrected to 11.5 mmHg so that the final sample size would be 200. The correction does not influence the protocol design and the conclusion of the study.
